# Efficient access to *N*-trifluoroacetylated 2′-amino-2′-deoxyadenosine phosphoramidite for RNA solid-phase synthesis

**DOI:** 10.1007/s00706-019-02390-x

**Published:** 2019-04-29

**Authors:** Christoph Falschlunger, Ronald Micura

**Affiliations:** Institute of Organic Chemistry, Leopold-Franzens University, Innrain 80-82, Innsbruck, Austria

**Keywords:** Nucleosides, Modifications, Oligoribonucleotides, RNA structure, Hydrogen bonding, Pistol ribozyme

## Abstract

**Abstract:**

Here, we present a robust synthetic route to a 2′-amino-2′-deoxyadenosine phosphoramidite building block for automated RNA solid-phase synthesis. The thus accessible 2′-amino-modified RNA finds applications in the evaluation of hydrogen-bond networks in folded RNA, such as riboswitches or ribozymes. In this context, we previously implemented the here described 2′-amino-2′-deoxyadenosine building block in a comparative study on self-cleaving pistol ribozymes to shed light on structural versus catalytic roles of active-site 2′-OH groups in the reaction mechanism.

**Graphical abstract:**

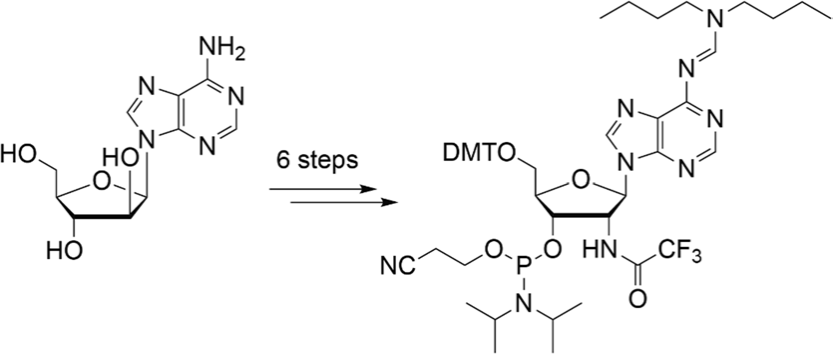

**Electronic supplementary material:**

The online version of this article (10.1007/s00706-019-02390-x) contains supplementary material, which is available to authorized users.

## Introduction

Several reports on the syntheses and properties of 2′-amino-2′-deoxy-modified RNA exist in the literature; however, most of them refer to 2′-amino-2′-deoxy pyrimidine nucleoside containing RNA only [1–8]. While access to 2′-amino-2′-deoxyuridine and 2′-amino-2′-deoxycytidine building blocks is straightforward (based on the key intermediate of 2,2′-anhydrouridine) [1], access to the corresponding 2′-aminoadenosine and 2′-aminoguanosine derivatives is more troublesome and in general requires multiple steps. In 1979, Eckstein and coworkers reported the synthesis of 2′-amino-2′-deoxyribofuranosyl purines based on trimethylsilyl trifluoromethanesulfonate-catalyzed transglycosylation reactions using *N*-trifluoroacetylated 2′-amino-2′-deoxyuridine and *N*-acylated purines [2]. The extension of this approach towards the *N*-trifluoroacetylated 2′-amino-2′-deoxyguanosine phosphoramidite was mentioned by the same authors in an investigation on the catalytic activity of the hammerhead ribozyme [3]. In 1998, Pfleiderer reported on the synthesis of 2′-amino-2′-deoxyadenosine and guanosine 3′-*O* phosphoramidites for oligonucleotide synthesis with the aglycone and the 2′-amino functions protected by the 2-(4-nitrophenyl)ethoxycarbonyl (npeoc) group [9]. The corresponding RNAs require customized deprotection via ß-elimination using 1,8-diazabicyclo[5.4.0]undec-7-en (DBU). In 2007, Richert introduced *N*,*O*- allyloxycarbonyl(alloc)-protected 5′-*O* phosphoramidites of 2′-amino-2′-deoxyadenosine for orthogonal protection schemes to access 2′-acylamido caps of DNA duplexes [10]. At the same time, an interesting concept for the prefunctionalization of nucleic acids based on 2′-methoxalylamino-2′-deoxyadenosine phosphoramidites was reported by Vasileva [11]. Finally, Beigelman and coworkers described in detail the synthesis of an *N*-(4-*tert.*-butylbenzoyl)-2′-deoxy-2′-*N*-phthaloyladenosine building block on a 100 g scale [7]. Later on, our group refined this path to prepare *N*-acetylated 2′-deoxy-2′-*N*-phthaloyladenosine phosphoramidite that was utilized for atomic mutagenesis in reconstituted ribosomes to explore the catalytic mechanism of ribosomal peptide bond formation [8]. A disadvantage of the 2′-*N*-phthaloyl protection, however, refers to our observation that it is rather stable and not completely removed under “ultramild” RNA deprotection conditions. Such conditions, however, are required for the preparation of long (> 40 nucleotides) synthetic RNA. Therefore, we elaborated the synthesis of the *N*-trifluoroacetylated 2′-amino-2′-deoxyadenosine phosphoramidite **6** as described below.

## Results and discussion

Our synthetic route started with the simultaneous protection of the 5′- and 3′-hydroxyl groups of commercially available 9-(*ß*-d-arabinofuranosyl)adenine using 1,3-dichloro-1,1,3,3-tetraisopropyldisiloxane (TIPDSCl_2_) to furnish nucleoside derivative **1** (Scheme 1) [12]. After triflation of the arabinose 2′-OH, the compound was reacted with sodium azide, producing compound **2** [13]. Then, the 2′-azido group of **2** was reduced by hydrogenation (H_2_ balloon) under Pd/C catalysis. After work-up, the resulting amino group was protected by trifluoroacetylation applying a two-step procedure involving ethyltrifluoroacetate first, followed by trifluoroacetic anhydride to increase the overall yields of compound **3**. Protection of the exocyclic adenine 6-amino group was achieved using *N*,*N*-dibutylformamide dimethylacetal (prepared as described in “Experimental” [14–17]), and subsequently, the TIPDS moiety was deprotected using tetrabutylammonium fluoride (TBAF) and acetic acid to yield compound **4**. Finally, this nucleoside was transformed into the dimethoxytritylated derivative **5** under standard conditions. The conversion of **5** into the corresponding phosphoramidite **6** was achieved in high yields by treatment with 2-cyanoethyl *N*,*N*-diisopropylchlorophosphoramidite under basic conditions. Starting with arabinoadenosine, our route provides **6** in 23% overall yield in six steps with seven chromatographic purifications; in total, 500 mg of phosphoramidite **6** was obtained in the course of this study.

**Figure:**
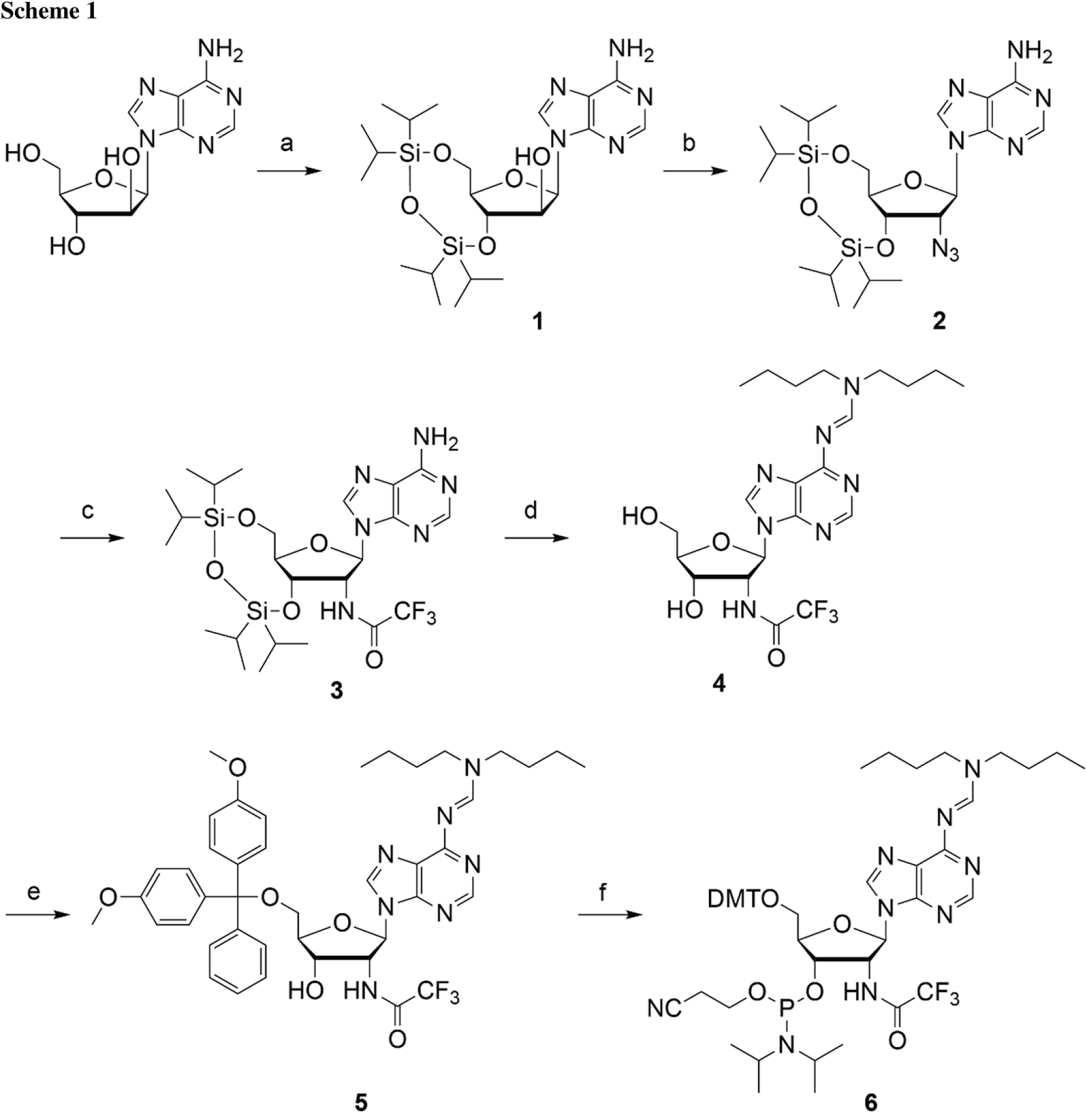
Reaction conditions: **a** 1.3 equiv 1,3-dichloro-1,1,3,3-tetraisopropyldisiloxane (TIPSiCl_2_) in anhydrous DMF and pyridine, room temperature, 14 h, 81%; **b** i) 1.5 equiv CF_3_SO_2_Cl, 3 equiv 4-(dimethylamino)pyridine (DMAP) in CH_2_Cl_2_, 0°C, 30 min; ii) 5 equiv NaN_3_ in DMF, room temperature, 15 h, 75%; **c** i) palladium on carbon, H_2_ (g) in THF, room temperature, overnight; ii) 10 equiv ethyltrifluoroacetate, 1.0 equiv trifluoroacetic anhydride in THF, room temperature, 48 h, 71%; **d** i) 3 equiv Bu_2_NCH(OCH_3_)_2_ [14–17] in THF, 60°C, overnight; ii) 1 M TBAF, 0.5 M acetic acid in THF, room temperature, 2 h, 78%; **e** 1.3 equiv 4,4'-dimethoxytrityl chloride (DMT-Cl), 0.3 equiv 4-(dimethylamino)pyridine (DMAP) in pyridine, room temperature, overnight, 81%; **f** 2 equiv* N,N*-diisopropylethylamine, 1.5 equiv 2-cyanoethyl* N,N*-diisopropylchlorophosphoramidite (CEP-Cl) in dichloromethane, room temperature, 2 h, 85%; total yield over six steps: 23%

## Conclusion

Our motivation to synthesize *N*-trifluoroacetylated 2′-amino-2′-deoxyadenosine phosphoramidite derivative **6** originated from our interest in the elucidation of ribozyme mechanisms. This particular building block **6** was utilized in a comprehensive study on pistol ribozymes [18], a recently discovered novel class of ribozymes that site-specifically cleaves their own phosphodiester backbone, resulting in two defined RNA fragments [19–21]. That study revealed the structural rather than catalytic role of a 2′-OH group of an active-site adenosine (A32), based on cleavage assays that allowed a comparison of cleavage activities of the wild-type ribozyme and the 2′-NH_2_ modified counterpart at varying pH values [18].

## Experimental

Reagents were purchased in the highest available quality from commercial suppliers (Sigma-Aldrich, abcr) and used without further purification. Moisture sensitive reactions were carried out under argon atmosphere. ^1^H and ^13^C spectra were recorded on a Bruker DRX 300 MHz spectrometer. Chemical shifts (*δ*) are reported relative to tetramethylsilane (TMS) referenced to the residual proton signal of the deuterated solvent (DMSO-*d*_*6*_: 2.50 ppm for ^1^H spectra and 39.52 ppm for ^13^C spectra). The following abbreviations were used to denote multiplicities: *s* = singlet, *d* = doublet, *t* = triplet, *m* = multiplet, and *b* = broad. Signal assignments are based on ^1^H-^1^H-COSY, ^1^H-^13^C-HSQC, and ^1^H-^13^C-HMBC experiments. MS experiments were performed on a Thermo Scientific Q Exactive Orbitrap with an electrospray ion source. Samples were analyzed in the positive ion mode. Reaction control was performed via analytical thin-layer chromatography (TLC, Macherey–Nagel) with fluorescent indicator. Spots were further visualized using cerium molybdate or anisaldehyde staining reagents. Column chromatography was carried out on silica gel 60 (70–230 mesh).

### *N*,*N*-Dibutylformamide dimethylacetal (C_11_H_25_NO_2_)

[in analogy to references 14,15] *N*,*N*-Dibutylformamide (21.6 g, 25 cm^3^, 0.14 mmol) was treated with 17.7 g dimethyl sulfate (13.2 cm^3^, 0.14 mmol) and heated to 100 °C for 4 h. The mixture was allowed to cool to room temperature and stirring was continued overnight. Sodium (4 g, 0.17 mmol) was dissolved in 75 cm^3^ absolute methanol and the solution cooled to 0 °C in an ice bath. Subsequently, the cold alcoholate solution was slowly added to the vigorously stirred reaction mixture and a white precipitate formed. After the suspension reached ambient temperature, diethyl ether was added and the precipitate was filtered off. All solvents were evaporated and the crude product was subjected to high vacuum fractional distillation (Vigreux column, b.p.: 54 °C [2 × 10^−2^ mbar]) to yield 14.5 g (51%) of a clear colorless oil. ^1^H NMR (400 MHz, CDCl_3_): *δ* = 0.88 (t, *J* = 7.3 Hz, 6H, C*H*_3_CH_2_CH_2_CH_2_N), 1.27 (dd, *J* = 7.2, 7.7 Hz, 4H, (CH_3_C*H*_*2*_CH_2_CH_2_)_2_N), 1.38 (m, 4H, (CH_3_CH_2_C*H*_*2*_CH_2_)_2_N), 2.57 (dd, *J* = 7.2, 7.7 Hz, 2H, ((CH_3_CH_2_CH_2_C*H*_*2*_)_2_N), 3.28 (s, 6H, (OCH_3_)), 4.49 (s, 1H, CH) ppm; ^13^C NMR (101 MHz, CDCl_3_): *δ* = 14.09 ((*C*H_3_CH_2_CH_2_CH_2_)_2_N), 20.63 ((CH_3_*C*H_2_CH_2_CH_2_)_2_N), 31.12 ((CH_3_CH_2_*C*H_2_CH_2_)_2_N), 47.21 ((CH_3_CH_2_CH_2_*C*H_2_)_2_N), 53.92 (CH_3_O), 112.79 (N–CH) ppm.

### 9-[3′,5′-*O*-(1,1,3,3-Tetraisopropyldisiloxane-1,3-diyl)-*β*-d-arabinofurano-1-syl]adenine (1, C_22_H_39_N_5_O_5_Si_2_)

[in analogy to reference 12] 9-*β*-d-Arabinofuranosyladenine (2.00 g, 7.5 mmol) was coevaporated three times with anhydrous pyridine. The residue was suspended in a 1:1 mixture of 16 cm^3^ anhydrous pyridine and *N*,*N*-dimethylformamide. Subsequently, 3.07 g 1,3-dichloro-1,1,3,3-tetraisopropyldisiloxane (3.11 cm^3^, 9.7 mmol) was added dropwise and stirred at room temperature for 24 h. The suspension turned into a colorless solution and all volatiles were removed by high vacuum distillation at 40 °C. The crude product was dissolved in methylene chloride and consecutively extracted with a solution of 5% sodium bicarbonate and saturated sodium chloride. After the organic phase was dried and evaporated, column chromatography (silica gel, 0 to 8% methanol in methylene chloride) was applied providing 3.07 g (81%) of compound **1** as white foam. TLC (10% methanol in methylene chloride): *R*_*f*_ = 0.49; ^1^H NMR (300 MHz, DMSO-*d*_*6*_): *δ* = 1.02–1.05 (m, 24H, (C*H*_3_)_2_CHSi), 1.10–1.14 (m, 4H, (CH_3_)_2_C*H*Si), 3.77–3.82 (m, 1H, H-C(4′)), 3.92 (dd, *J* = 2.6, 12.4 Hz, 1H, H(a)-C(5′)), 4.10 (dd, *J* = 4.4, 12.6 Hz, 1H, H(b)-C(5′)), 4.50 (dd, *J* = 7.3, 13.7 Hz, 1H, H-C(2′)), 4.57 (dd, *J* = 7.7, 15.6 Hz, 1H, H-C(3′)), 5.78 (d, *J* = 5.7 Hz, 1H, HO-C(2′)), 6.20 (d, *J* = 6.4 Hz, 1H, H-C(1′)), 7.27 (s, 2H, H-N(6)), 8.04 (s, 1H, H-C(2)), 8.11 (s, 1H, H-C(8)) ppm; ^13^C NMR (75 MHz, DMSO-*d*_*6*_): *δ* = 11.99–12.83 ((CH_3_)_2_*C*HSi), 16.79–17.35 ((*C*H_3_)_2_CHSi), 61.51 (C(5′)), 74.89 (C(2′/3′)), 75.14 (C(2′/3′)), 79.59 (C(4′)), 81.83 (C(1′)), 118.52 (C(5)), 139.61 (C(8)), 149.55 (C(4)), 152.38 (C(2)), 156.00 (C(6)) ppm; ESI–MS: *m/z* calculated for [C_22_H_40_N_5_O_5_Si_2_]^+^ ([M + H]^+^): 510.2528, found 510.2562.

### 2′-Azido-2′-deoxy-3′,5′-*O*-(1,1,3,3-tetraisopropyldisiloxane-1,3-diyl)adenosine (2, C_22_H_38_N_8_O_4_Si_2_)

[in analogy to reference 13] Compound **1** (3.07 g, 6.0 mmol) and 2.2 g 4-(dimethylamino)pyridine (18 mmol) were dissolved in 25 cm^3^ anhydrous methylene chloride and the resulting solution was cooled to 0 °C. Trifluoromethanesulfonyl chloride (1.52 g, 0.96 cm^3^, 9 mmol) was added in one portion to the stirred reaction mixture. The solution turned yellow and stirring was continued for 30 min. By adding a solution of 5% sodium bicarbonate solution, the reaction was quenched and extracted at once. The organic phase was dried, evaporated, and dissolved in 25 cm^3^ anhydrous *N*,*N*-dimethylformamide. The solution was treated with 1.95 g sodium azide (30 mmol) and stirred at room temperature for 15 h. All volatiles were removed under high vacuum at 40 °C and the residue was dissolved in methylene chloride. Consecutively, the solution was extracted with an aqueous solution of 5% citric acid and 5% sodium bicarbonate, dried, and evaporated to dryness. Column chromatography (silica gel, 0–1.5% methanol in methylene chloride) afforded 2.41 g (75%) of compound **2** as slightly yellow foam. TLC (10% methanol in methylene chloride): *R*_*f*_ = 0.56; ^1^H NMR (300 MHz, DMSO-*d*_*6*_): *δ* = 1.02–1.11 (m, 28H, (C*H*_3_)_2_C*H*Si), 3.92–4.00 (m, 3H, H-C(4′,5′)), 5.01 (d, *J* = 5.8 Hz, 1H, H-C(2′)), 5.44 (dd, *J* = 6.04, 7.8 Hz, 1H, H-C(3′)), 5.83 (s, 1H, H-C(1′)), 7.35 (s, 2H, H-N(6)), 8.06 (s, 1H, H-C(2)), 8.22 (s, 1H, H-C(8)) ppm; ^13^C NMR (75 MHz, DMSO-*d*_*6*_): *δ* = 12.71–13.30 ((CH_3_)_2_*C*HSi), 17.33–17.86 ((*C*H_3_)_2_CHSi), 61.42 (C(5′)), 65.23 (C(3′)), 72.50 (C(2′)), 81.60 (C(4′)), 86.77 (C(1′)), 119.94 (C(5)), 140.55 (C(8)), 149.12 (C(4)), 153.03 (C(2)), 156.76 (C(6)) ppm; ESI–MS: *m/z* calculated for [C_22_H_39_N_8_O_4_Si_2_]^+^ ([M + H]^+^): 535.2633, found 535.2649.

### 2′-Deoxy-3′,5′-*O*-(1,1,3,3-tetraisopropyldisiloxane-1,3-diyl)-2′-[(trifluoroacetyl)amino]adenosine (3, C_24_H_39_F_3_N_6_O_5_Si_2_)

Compound **2** (711 mg, 1.33 mmol) and palladium on activated charcoal (75 mg) were suspended with 25 cm^3^ tetrahydrofuran. The suspension was treated with hydrogen and stirred under hydrogen atmosphere overnight. Subsequently, the charcoal was removed by filtration over Celite, the solution evaporated, and the residue dissolved in 10 cm^3^ tetrahydrofuran. Ethyltrifluoroacetate (1.89 g, 1.58 cm^3^, 13.3 mmol) was added to the solution and stirring was continued for 24 h. The reaction mixture was evaporated and the residue subjected to column chromatography (silica gel, 2–10% methanol in methylene chloride) to afford 265 mg of compound **3** and a mixture of **3** and starting material. The latter was dissolved in 20 cm^3^ tetrahydrofuran, followed by the addition of 277 mg trifluoroacetic anhydride (0.183 cm^3^, 1.32 mmol). The reaction mixture was stirred for another 24 h, evaporated, extracted with a solution of 5% sodium bicarbonate in water, and dried over sodium sulfate. After evaporation, the residue was subjected to column chromatography (silica gel, 0–5% methanol in methylene chloride). In total, 568 mg (71%) of compound **3** were obtained as slightly pink foam. TLC (10% methanol in methylene chloride): *R*_*f*_ = 0.51; ^1^H NMR (300 MHz, DMSO-*d*_*6*_): *δ* = 1.00–1.10 (m, 28H, (C*H*_3_)_2_C*H*Si), 3.96–3.97 (m, 2H, H-C(5′)), 4.03–4.09 (m, 1H, H-C(4′)), 5.10 (dd, *J* = 7.4, 7.8 Hz, 1H, H-C(3′)), 5.20–5.26 (m, 1H, H-C(2′)), 6.11 (d, *J* = 3.6 Hz, 1H, H-C(1′)), 7.36 (s, 2H, H-N(6)), 8.09 (s, 1H, H-C(2)), 8.34 (s, 1H, H-C(8)), 9.86 (d, *J* = 8.9 Hz, 1H, H-N(2′)) ppm; ^13^C NMR (75 MHz, DMSO-*d*_*6*_): *δ* = 12.14–12.64 ((CH_3_)_2_*C*HSi), 16.64–17.29 ((*C*H_3_)_2_CHSi), 55.09 (C(2′)), 62.26 (C(5′)), 70.23 C(3′)), 82.34 (C(4′)), 85.56 (C(1′)), 119.25 (C(5)), 140.22 (C(8)), 148.81 (C(4)), 152.61 (C(2)), 156.20 (C(6)) ppm; ESI–MS: *m/z* calculated for [C_24_H_40_F_3_N_6_O_5_Si_2_]^+^ ([M + H]^+^): 605.2551, found 605.2526.

### 2′-Deoxy-*N*^*6*^-[(dibutylamino)methylidene]-2′-[(trifluoroacetyl)amino]adenosine (4, C_21_H_30_F_3_N_7_O_4_)

*N*,*N*-Dibutylformamide dimethyl acetal (1.29 g, 1.46 cm^3^, 6.33 mmol; prepared as described above) was added to a solution of 1.28 g of compound **3** (2.11 mmol) in 20 cm^3^ anhydrous tetrahydrofuran. The reaction was stirred overnight at 60 °C followed by evaporation. The residue was dissolved in a solution of 1 M tetrabutylammonium fluoride trihydrate and 0.5 M acetic acid in tetrahydrofuran (8.4 cm^3^). After 2 h of stirring the reaction, all volatiles were removed and the crude product was extracted with saturated sodium bicarbonate. The organic layer was evaporated and subjected to column chromatography (silica gel, 0–10% methanol in methylene chloride) giving a yield of 825 mg (78%) white foam. TLC (10% methanol in methylene chloride): *R*_*f*_ = 0.46; ^1^H NMR (300 MHz, DMSO-*d*_*6*_): *δ* = 0.89–0.95 (m, 6H, C*H*_3_CH_2_CH_2_CH_2_N), 1.27-1.37 (m, 4H, (CH_3_C*H*_*2*_CH_2_CH_2_)_2_N), 1.54–1.66 (m, 4H, (CH_3_CH_2_C*H*_*2*_CH_2_)_2_N), 3.45 (dd, *J* = 7.5, 6.4 Hz, 2H, ((CH_3_CH_2_CH_2_C*H*_*2*_)_2_N(b)), 3.59 (dd, *J* = 7.1, 7.5 Hz, 2H, ((CH_3_CH_2_CH_2_C*H*_*2*_)_2_N(a)), H(b)-C(5′)), 3.68–3.75 (m, 2H, H(a)-C(5′)), 4.08 (m, 1H, H-C(4′)), 4.38 (m, 1H, H-C(3′)), 5.15 (m, 1H, H-C(2′)), 5.42 (dd, *J* = 5.4, 6.4 Hz, 1H, HO-C(5′)), 5.85 (d, *J* = 4.4 Hz, 1H, HO-C(3′)), 6.27 (d, *J* = 8.2 Hz, 1H, H-C(1′)), 8.37 (s, 1H, H-C(8)), 8.42 (s, 1H, H-C(2)), 8.92 (s, 1H, N(6)CH), 9.57 (d, *J* = 7.8 Hz, 1H, H-N(2′)) ppm; ^13^C NMR (75 MHz, DMSO-*d*_*6*_): *δ* = 13.55, 13.72 ((*C*H_3_CH_2_CH_2_CH_2_)_2_N), 19.15, 19.64 ((CH_3_*C*H_2_CH_2_CH_2_)_2_N), 28.68, 30.47 ((CH_3_CH_2_*C*H_2_CH_2_)_2_N), 44.51, 51.00 ((CH_3_CH_2_CH_2_*C*H_2_)_2_N), 55.53 (C(2′)), 61.76 (C(5′)), 69.77 (C(3′)), 85.10 (C(1′)), 87.18 (C(4′)), 125.98 (C(5)), 141.74 (C(8)), 151.12 (C(4)), 151.87 (C(2)), 158.00 (N(6)CH), 159.62 (C(6)) ppm; ESI–MS: *m/z* calculated for [C_21_H_31_F_3_N_7_O_4_]^+^ ([M + H]^+^): 502.2390, found 502.2385.

### 2′-Deoxy-*N*^*6*^-[(dibutylamino)methylidene]-5′-*O*-(4,4′-dimethoxytrityl)-2′-[(trifluoroacetyl)amino]adenosine (5, C_42_H_48_F_3_N_7_O_6_)

Compound **4** (390 mg, 0.78 mmol) was coevaporated four times with 5 cm^3^ anhydrous pyridine and dissolved in 10 cm^3^ thereof. To the orange solution, 342 mg 4,4′-dimethoxytrityl chloride (1.01 mmol) was added over a period of 1 h. Subsequently, the mixture was treated with 29 mg 4-(dimethylamino)pyridine (0.24 mmol) and stirring was continued overnight. After the reaction was quenched with 1 cm^3^ methanol, all volatiles were evaporated and the residue was dissolved in methylene chloride. The organic phase was extracted twice with an aqueous solution of 5% citric acid and saturated sodium bicarbonate. Column chromatography (silica gel, 0–5% methanol in methylene chloride) afforded 507 mg (81%) of compound **5** as slightly yellow foam. TLC (10% methanol in methylene chloride): *R*_*f*_ = 0.58; ^1^H NMR (300 MHz, DMSO-*d*_*6*_): *δ* = 0.86–0.97 (m, 6H, C*H*_3_CH_2_CH_2_CH_2_N), 1.26–1.37 (m, 4H, (CH_3_C*H*_*2*_CH_2_CH_2_)_2_N), 1.55–1.64 (m, 4H, (CH_3_CH_2_C*H*_*2*_CH_2_)_2_N), 3.29 (m, 2H, ^2^H-C(5′)), 3.44 (dd, *J* = 7.3, 6.7 Hz, 2H, (CH_3_CH_2_CH_2_C*H*_*2*_)_2_N(b)), 3.59 (dd, *J* = 7.1, 7.1 Hz, 2H, (CH_3_CH_2_CH_2_C*H*_*2*_)_2_N(a)), 4.17–4.21 (m, 1H, H-C(4′)), 4.51 (m, 1H, H-C(3′)), 5.38 (m, 1H, H-C(2′)), 6.28 (d, *J* = 7.1 Hz, 1H, H-C(1′)), 6.80–6.84 (m, 4H, H-C(trityl)), 7.20–7.40 (m, 9H, H-C(trityl)), 8.31 (s, 1H, H-C(2)), 8.33 (s, 1H, H-C(8)), 8.91 (s, 1H, N(6)CH), 9.67 (d, *J* = 8.9 Hz, 1H, H-N(2′)) ppm; ^13^C NMR (75 MHz, DMSO-*d*_*6*_): *δ* = 13.53,13.72 ((*C*H_3_CH_2_CH_2_CH_2_)_2_N), 19.12, 19.62 ((CH_3_*C*H_2_CH_2_CH_2_)_2_N), 28.67, 30.44 ((CH_3_CH_2_*C*H_2_CH_2_)_2_N), 44.46, 50.93 ((CH_3_CH_2_CH_2_*C*H_2_)_2_N), 54.67 (C(2′)), 54.96 (OCH_3_(trityl)), 63.70 (C(5′)), 69.23 (C(3′)), 84.53 (C(4′)), 85.61 (C(1′)), 113.11 (C(trityl)), 125.86 (C(5)), 126.65-129.75 (C(trityl)), 135.42, 135.46 (C(trityl)), 141.81 (C(8)), 144.82 (C(trityl)), 151.31 (C(4)), 151.98 (C(2)), 158.00 (N(6)CH), 141.81 (C(8)), 151.98 (C(2)) 157.85-158.07 (N(6)CHN, C(trityl)), 159.62 (C(6)) ppm; ESI–MS: *m/z* calculated for [C_42_H_49_F_3_N_7_O_6_]^+^ ([M + H]^+^): 804.3654, found 804.3691.

### 2′-Deoxy-*N*^*6*^-[(dibutylamino)methylidene]- 5′-*O*-(4,4′-dimethoxytrityl)-2′-[(trifluoroacetyl)amino]adenosine-3′-(2-cyanoethyl)-*N*,*N*-diisopropylphosphoramidite (6, C_51_H_65_F_3_N_9_O_7_P)

Compound **5** (206 mg, 0.26 mmol) was dissolved in 4 cm^3^ anhydrous methylene chloride. The solution was subsequently treated with 66 mg *N*,*N*-diisopropylethylamine (89 mm^3^, 0.51 mmol) and 91 mg 2-cyanoethyl *N*,*N*-diisopropylchlorophosphoramidite (86 mm^3^, 0.38 mmol). Stirring was continued for 2 h and the reaction was quenched by the addition of 1 cm^3^ methanol. The obtained mixture was extracted with a saturated solution of sodium bicarbonate in water, dried, and evaporated. Column chromatography (silica gel, 30–60% ethyl acetate in cyclohexane) gave 218 mg (85%) of compound **6** as white foam. TLC (10% methanol in methylene chloride): *R*_*f*_ = 0.81; ^1^H NMR (300 MHz, CDCl_3_): *δ* = 0.92–0.98 (m, 6H, C*H*_3_CH_2_CH_2_CH_2_)_2_N), 1.15–1.22 (m, 12H, ((C*H*_*3*_)_2_CH)_2_N), 1.33–1.43 (m, 4H, (CH_3_C*H*_*2*_CH_2_CH_2_)_2_N), 1.59-1.72 (m, 4H, (CH_3_CH_2_C*H*_*2*_CH_2_)_2_N), 2.42 (dt, *J* = 6.5, 6.2 Hz, 1H, C*H*_2_CN(b)), 2.60 (dd, *J* = 6.3, 6.2 Hz, 1H, C*H*_2_CN(a)), 3.32–3.53 (4H, (CH_3_CH_2_CH_2_C*H*_*2*_)_2_N(b), ^2^H-C(5′)), 3.54–3.73 (5H, (CH_3_CH_2_CH_2_C*H*_*2*_)_2_N(a), ((CH_3_)_2_C*H*)_2_N, POCH_2_(b)), 3.78 (s, 6H, OCH_3_(trityl)), 3.83–3.93 (1H, POCH_2_(a)), 4.36(a)/4.51(b) (m, 1H, H-C(4′)), 4.69 (dd, *J* = 5.5, 4.7 Hz, H-C(3′,a)), 4.83 (dd, *J* = 5.8, 4.7 Hz, H-C(3′,b)), 5.21 (dd, *J* = 6.9, 6.2 Hz, H-C(2′,a)), 5.28 (dd, *J* = 8.1, 6.3 Hz, H-C(2′,b)), 6.22 (dd, *J* = 11.0, 8.2 Hz, 1H, H-C(1′)), 6.80–6.83 (m, 4H, H-C(trityl)), 7.18–7.48 (m, 10H, H-C(trityl), H-N(2′)), 8.12, 8.18 (s, 1H, H-C(8)), 8.48, 8.49 (s, 1H, H-C(2)), 9.00 (s, 1H, N(6)CH) ppm; ^31^P NMR (121 MHz, CDCl_3_): *δ* = 151.09, 152.57 ppm; ESI–MS: *m/z* calculated for [C_51_H_66_F_3_N_9_O_7_P]^+^ ([M + H]^+^): 1004.4775, found 1004.4724.

## Electronic supplementary material

Below is the link to the electronic supplementary material.

Supplementary material 1 (PDF 1417 kb)
