# Supplementary material for: Efficient access to N-trifluoroacetylated 2′-amino-2′-deoxyadenosine phosphoramidite for RNA solid-phase synthesis
Source: Monatsh Chem. 2019 Apr 29;150(5):795–800. doi: 10.1007/s00706-019-02390-x (PMC6534076; doi:10.1007/s00706-019-02390-x)

Supporting Information

to

Efficient access to *N*-trifluoroacetylated 2'-amino-2'-deoxyadenosine phosphoramidite for RNA solid-phase synthesis

Christoph Falschlunger[1] ● Ronald Micura[1]

[1]Institute of Organic Chemistry and Center for Molecular Biosciences, University of Innsbruck, Austria

Contents

1. NMR spectra of *N*,*N*-dibutylformamide dimethylacetal 2
2. NMR spectra of compound **1** 3
3. NMR spectra of compound **2** 4
4. NMR spectra of compound **3** 5
5. NMR spectra of compound **4** 6
6. NMR spectra of compound **5** 7
7. NMR spectra of compound **6** 8

NMR spectra of *N*,*N*-dibutylformamide dimethylacetal

^{1}H-NMR (400 MHz, $CDCl_3$)

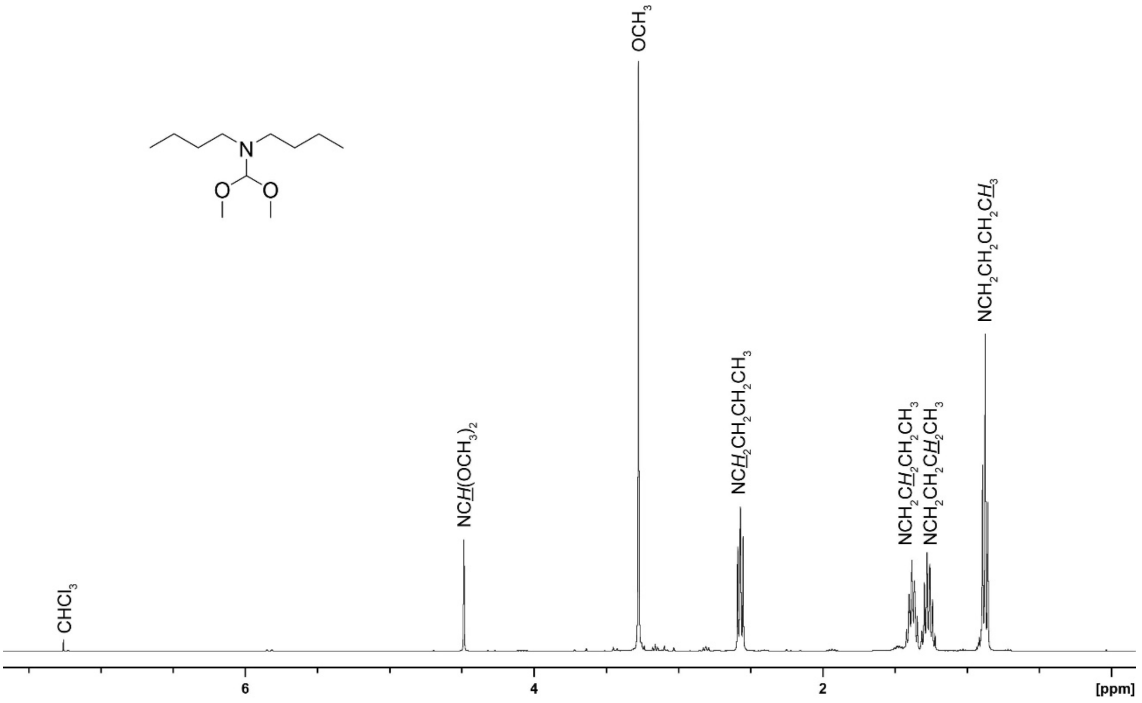

^{13}C-NMR (101 MHz, $CDCl_3$)

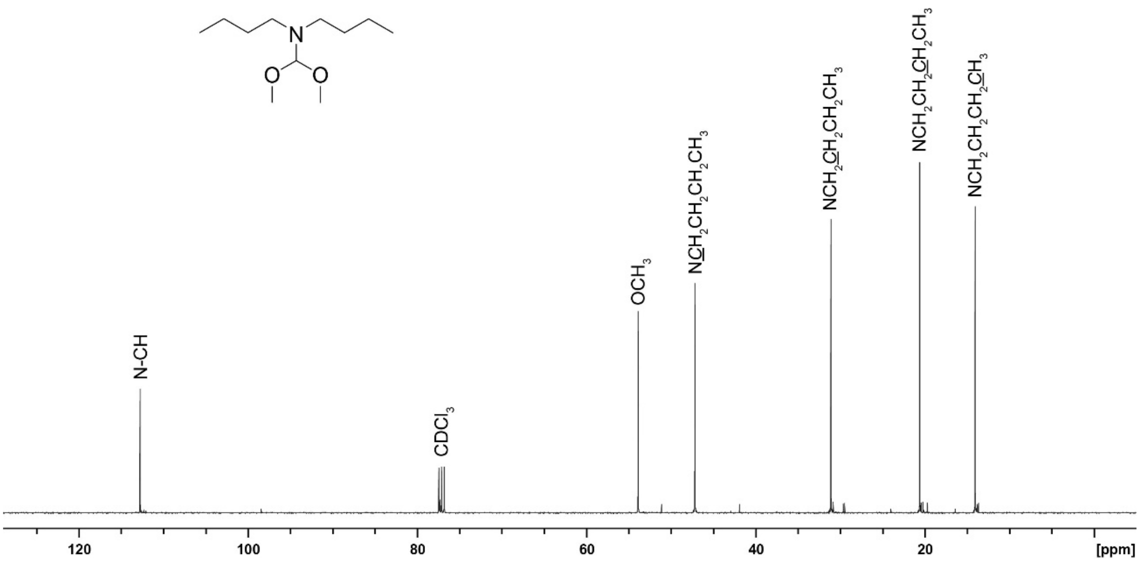

NMR spectra of compound 1

^{1}H-NMR (300 MHz, DMSO-d$_6$)

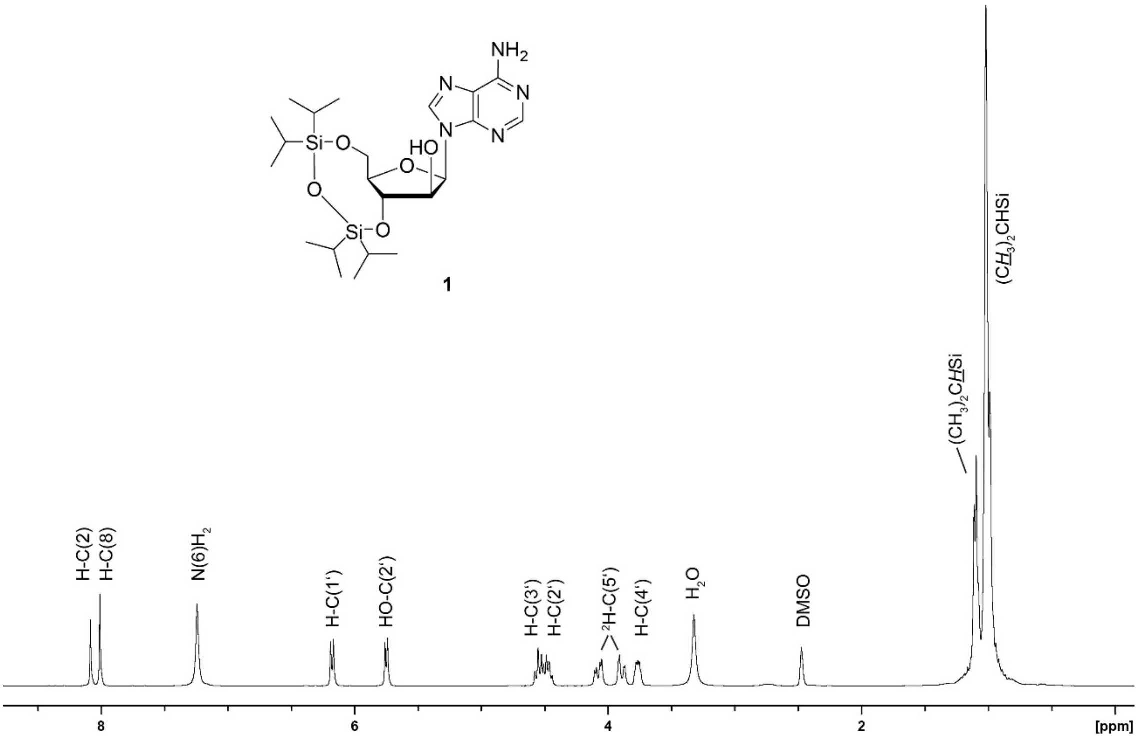

^{13}C-NMR (75 MHz, DMSO-d$_6$)

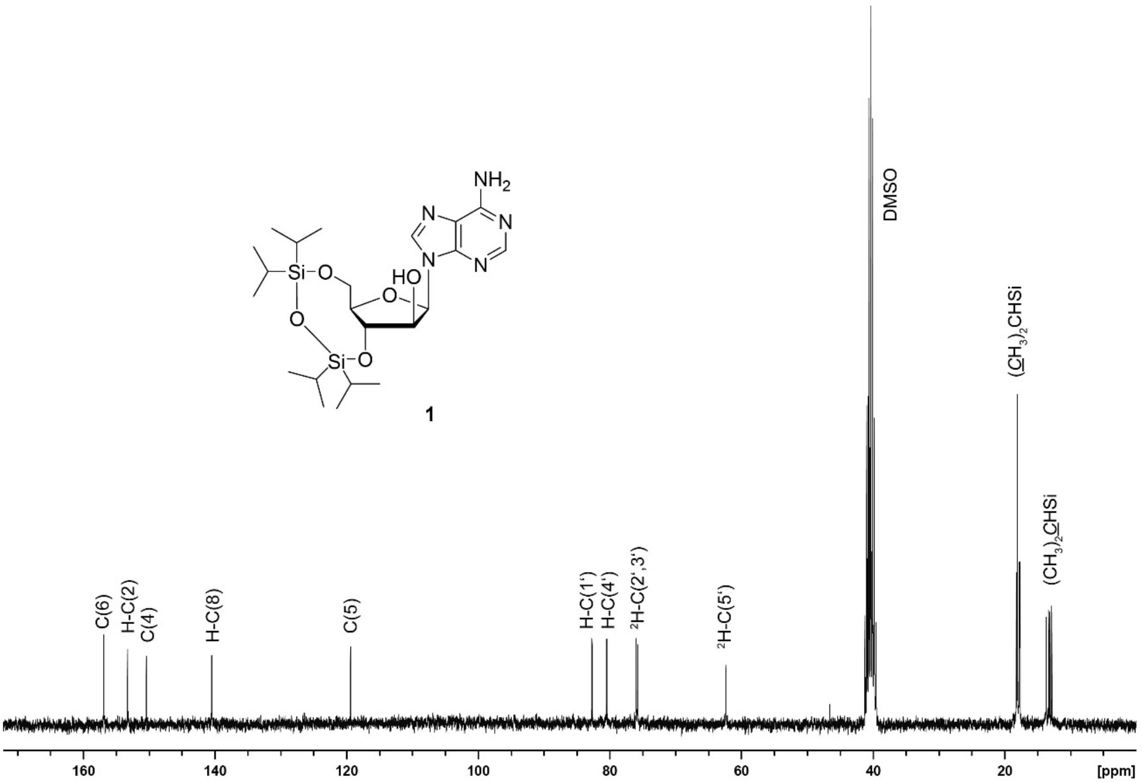

NMR spectra of compound 2

^{1}H-NMR (300 MHz, DMSO-d$_6$)

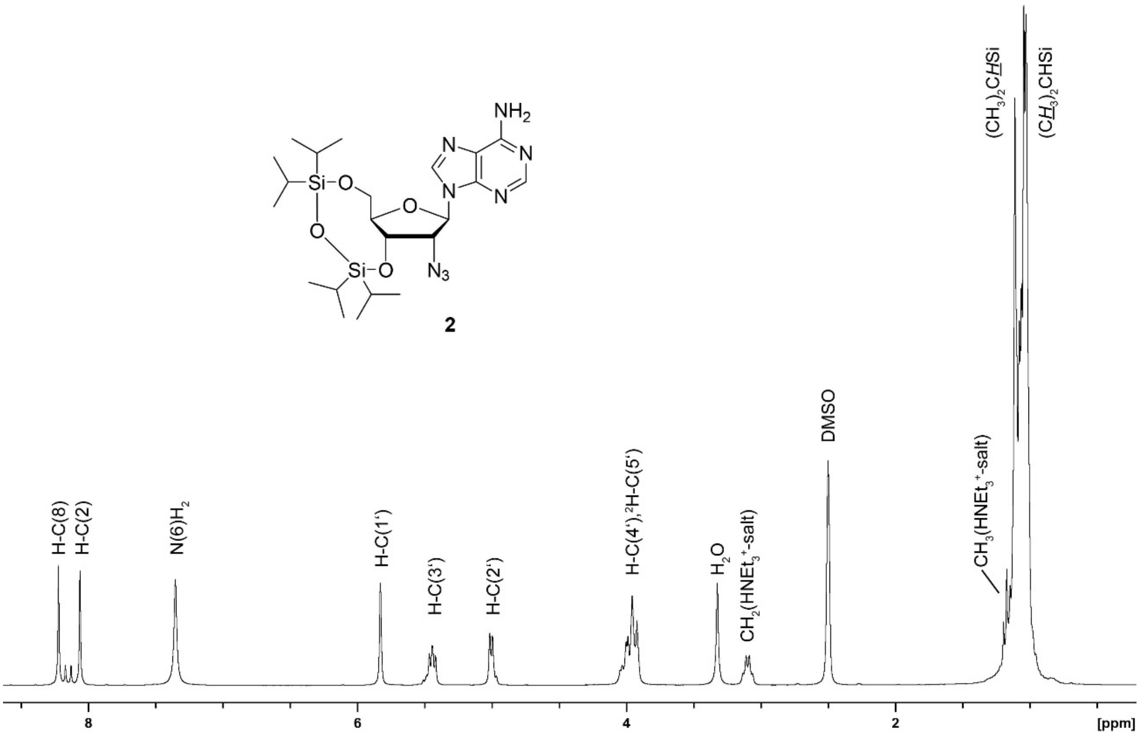

^{13}C-NMR (75 MHz, DMSO-d$_6$)

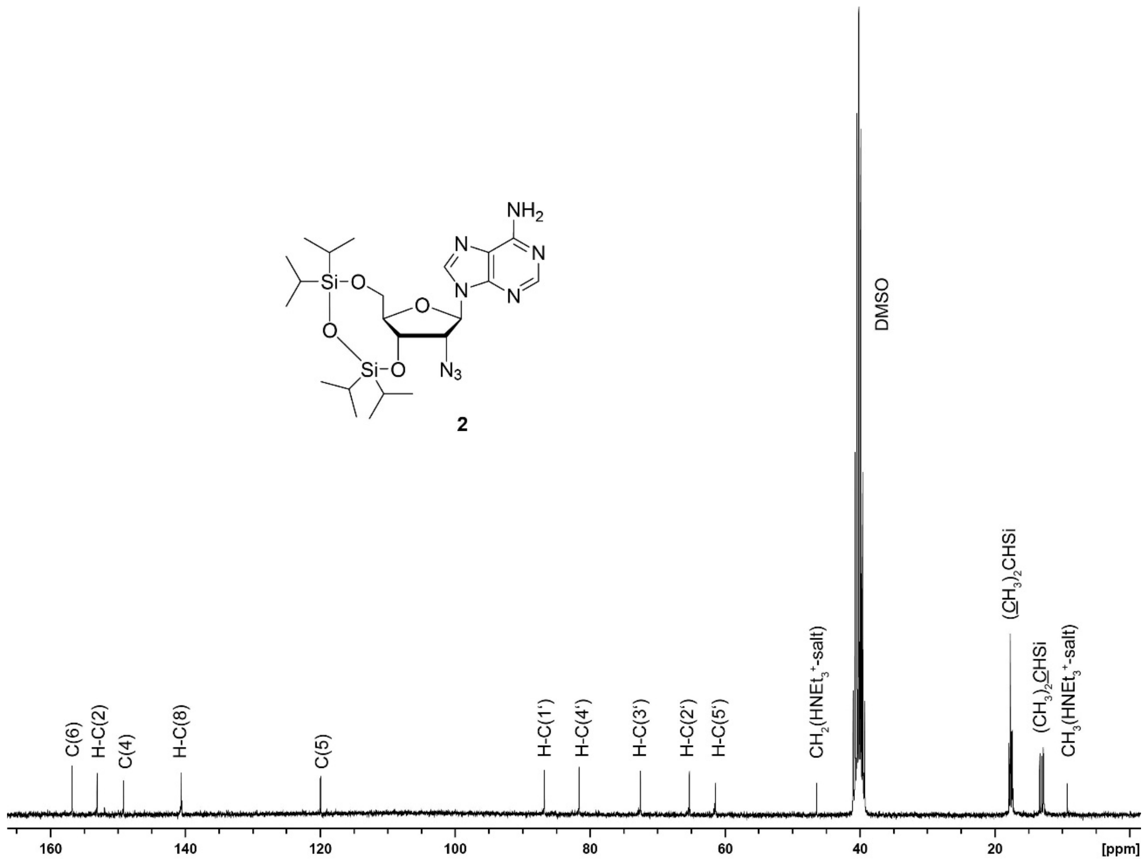

NMR spectra of compound 3

^{1}H-NMR (300 MHz, DMSO-d$_6$)

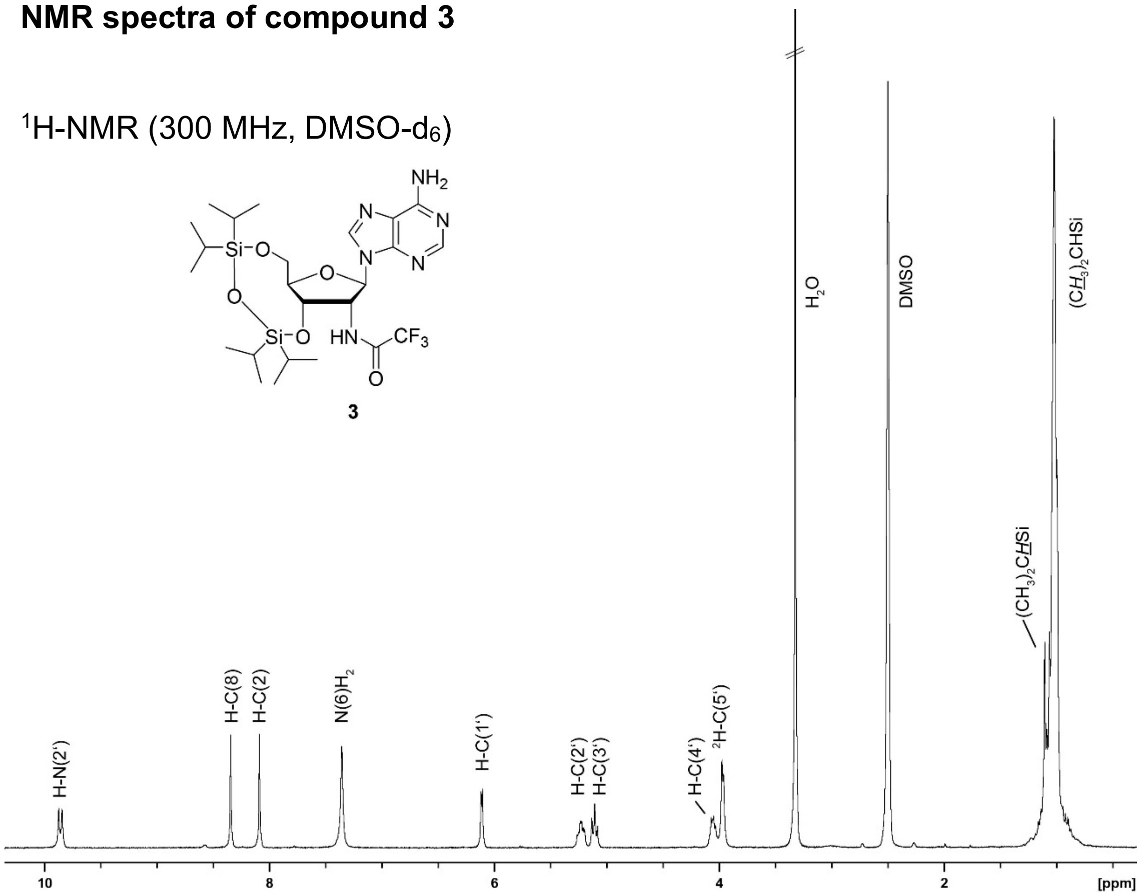

^{13}C-NMR (75 MHz, DMSO-d$_6$)

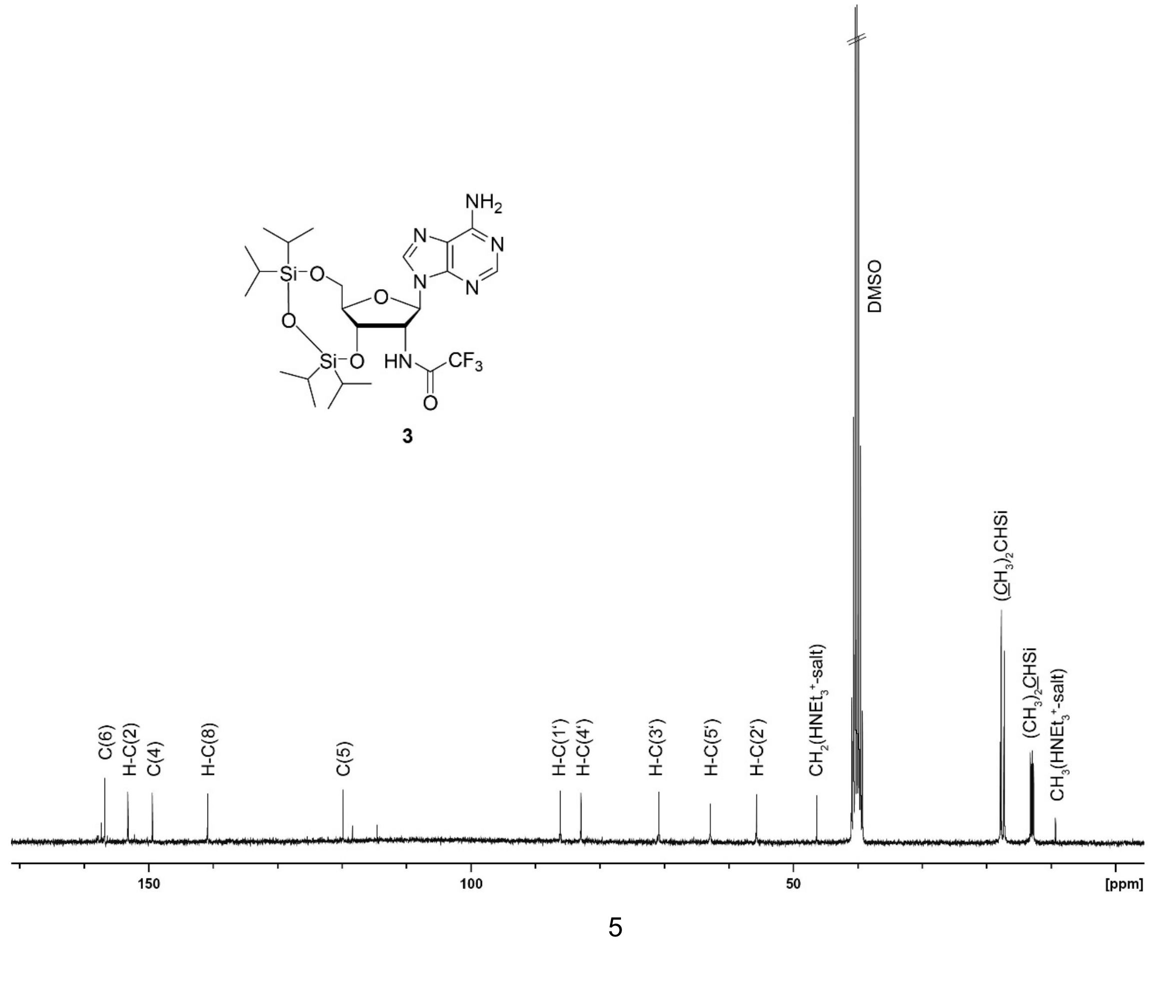

NMR spectra of compound 4

^{1}H-NMR (300 MHz, DMSO-d$_6$)

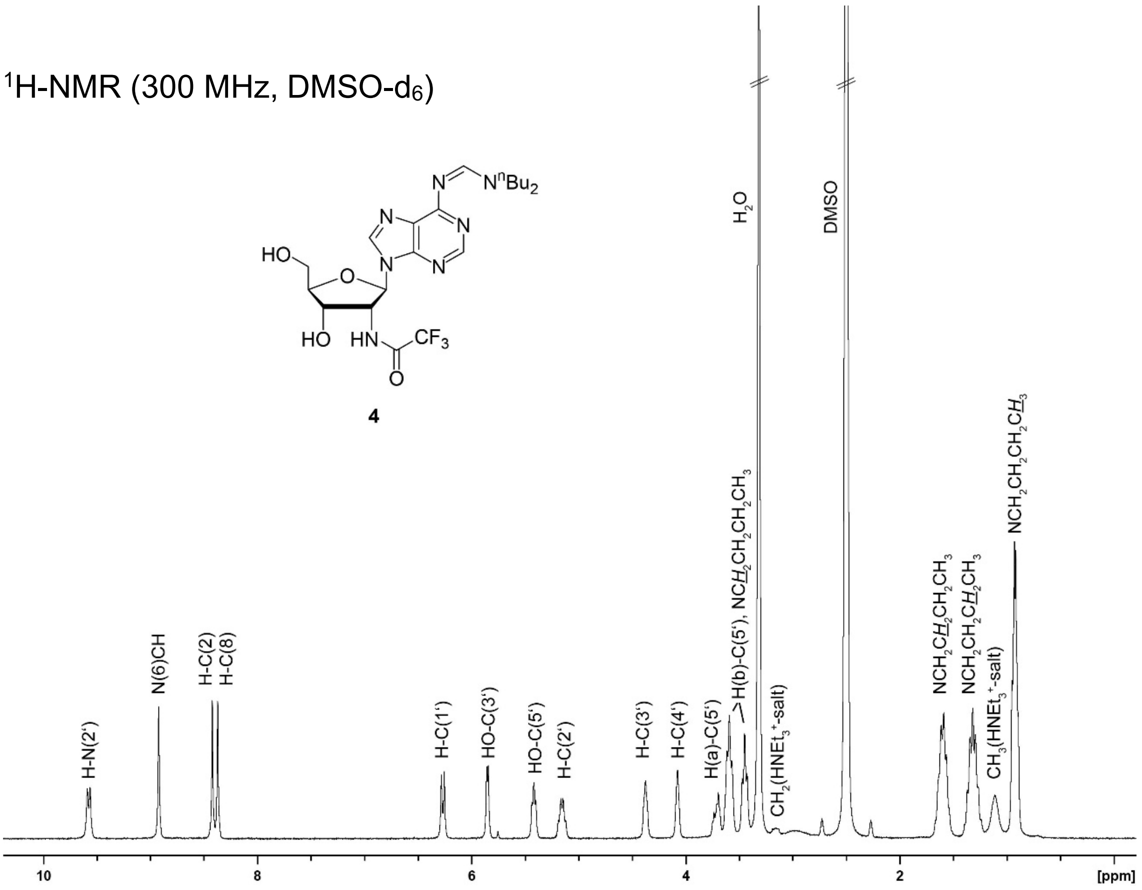

^{13}C-NMR (75 MHz, DMSO-d$_6$)

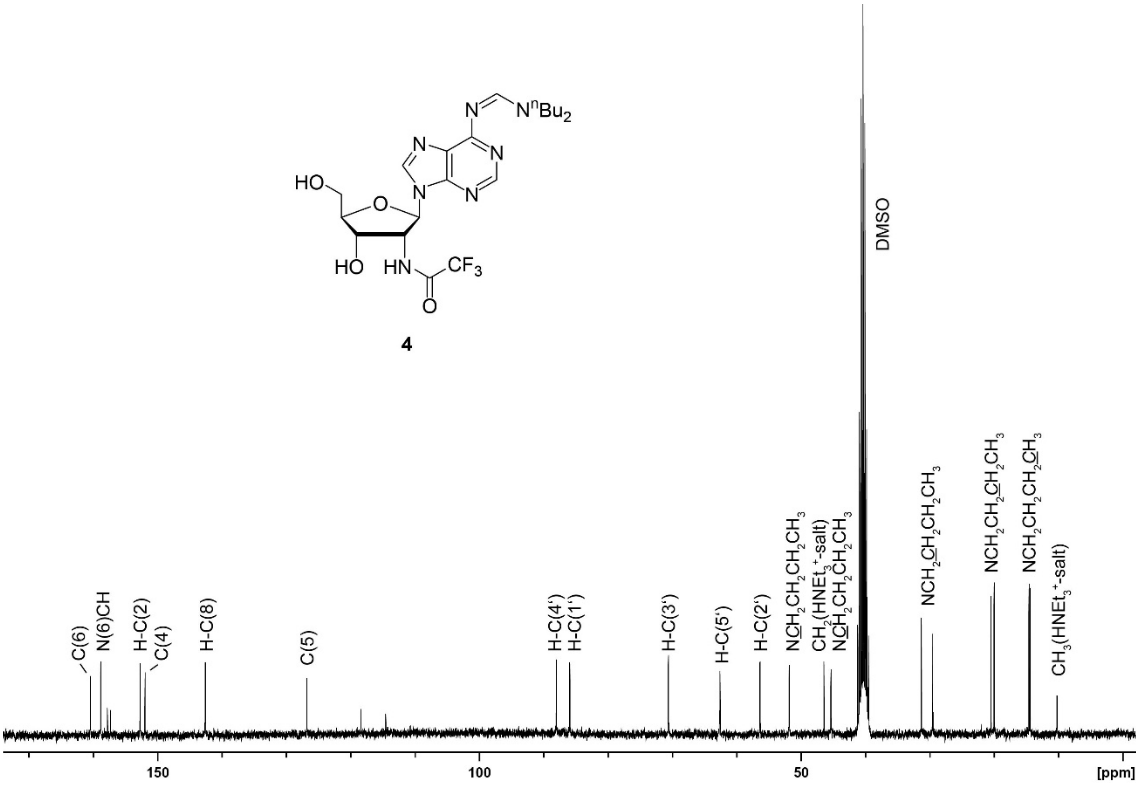

NMR spectra of compound 5

^{1}H-NMR (300 MHz, DMSO-d$_6$)

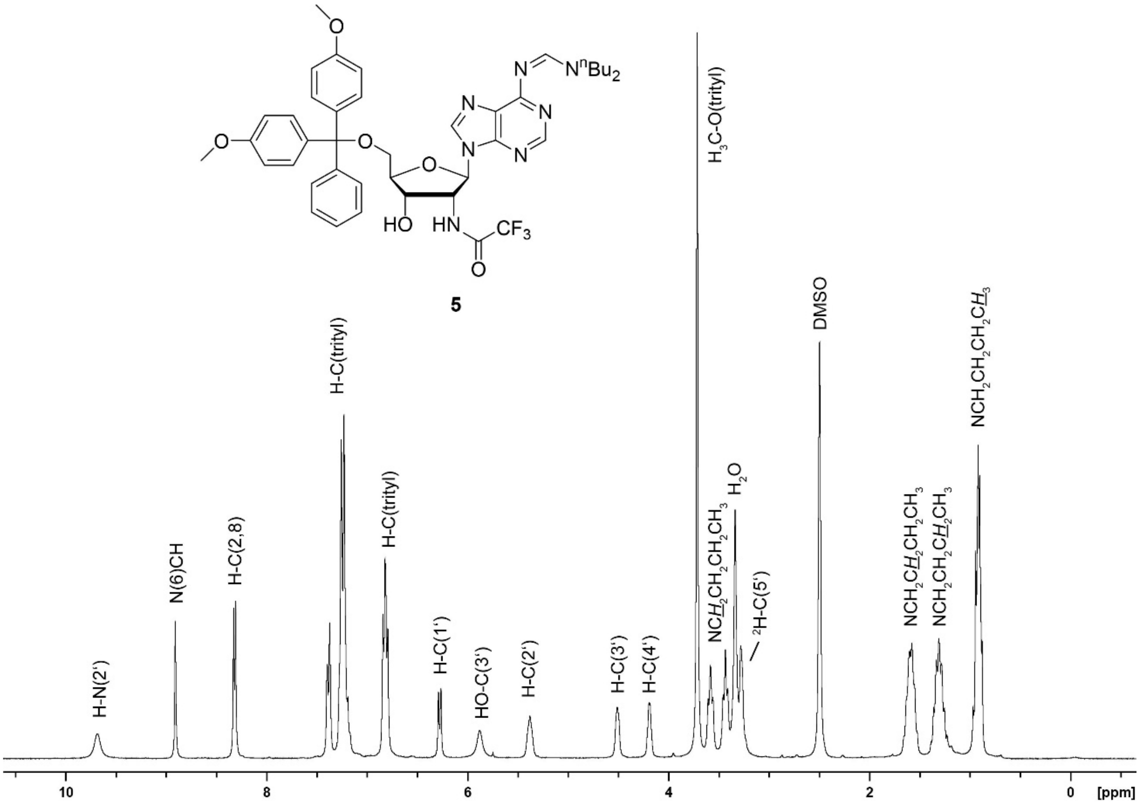

^{13}C-NMR (75 MHz, DMSO-d$_6$)

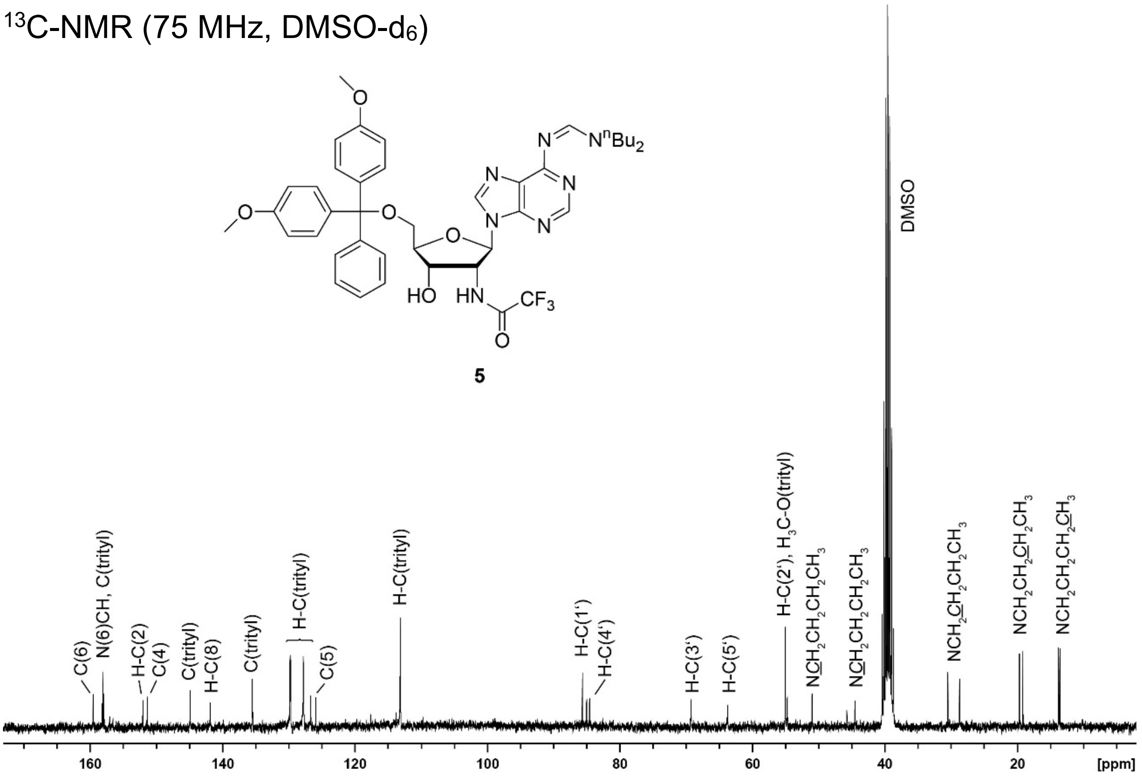

NMR spectra of compound 6

^{1}H-NMR (300 MHz, $CDCl_3$)

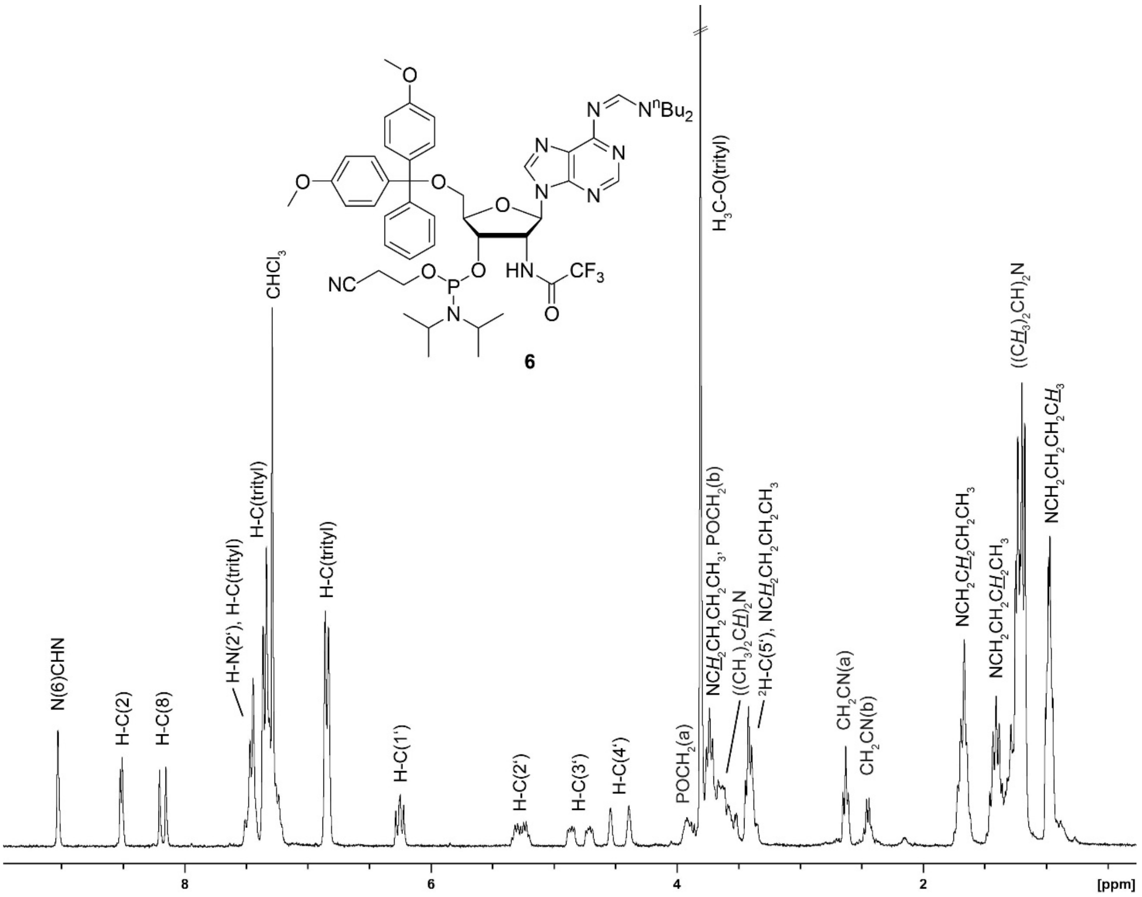

^{31}P-NMR (121 MHz, $CDCl_3$)

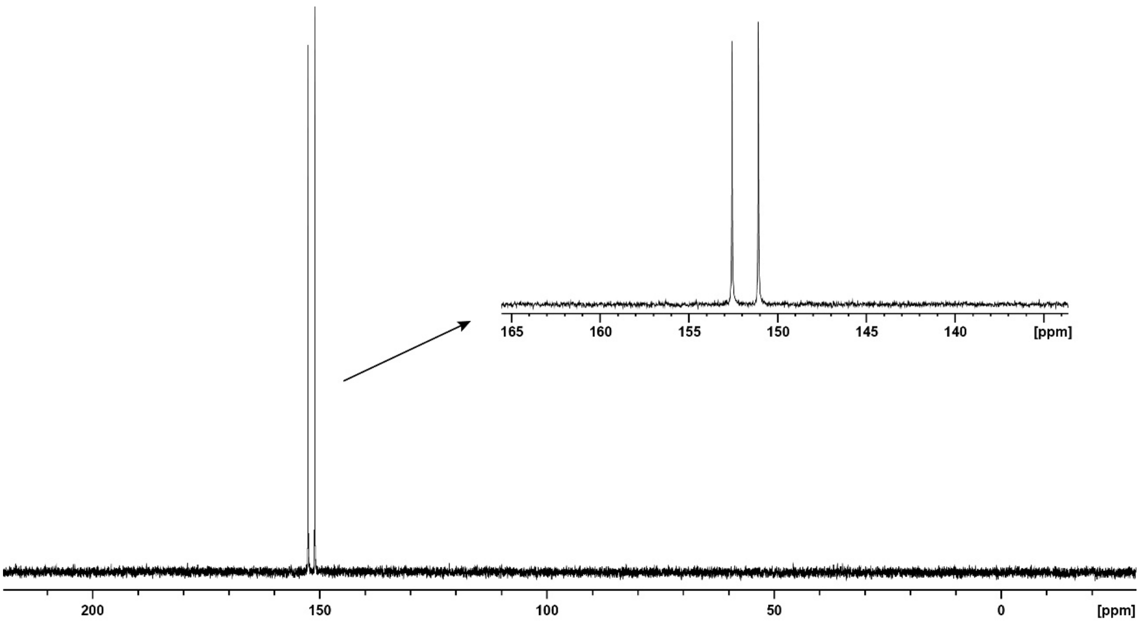

Supplement: Supplementary file 1 — Supplementary material 1 (PDF 1417 kb) [file 706_2019_2390_MOESM1_ESM.pdf]
